# Metabolomic and transcriptomic data on major metabolic/biosynthetic pathways in workers and soldiers of the termite *Prorhinotermes simplex* (Isoptera: Rhinotermitidae) and chemical synthesis of intermediates of defensive (*E*)-nitropentadec-1-ene biosynthesis

**DOI:** 10.1016/j.dib.2018.04.052

**Published:** 2018-05-02

**Authors:** Anna Jirošová, Andrej Jančařík, Riya C. Menezes, Olga Bazalová, Klára Dolejšová, Heiko Vogel, Pavel Jedlička, Aleš Buček, Jana Brabcová, Pavel Majer, Robert Hanus, Aleš Svatoš

**Affiliations:** a The Institute of Organic Chemistry and Biochemistry of the Czech Academy of Sciences, Flemingovo n. 2, 166 10 Prague, Czech Republic; b Czech University of Life Sciences Prague, Faculty of Forestry and Wood Sciences, Kamýcká 961/129, 165 00 Praha-Suchdol, Czech Republic; c Max-Planck Institute for Chemical Ecology, Hans-Knöll-Str. 8, 07745 Jena, Germany; d Biology Centre CAS, Branišovská 31, CZ-37005 České Budějovice, Czech Republic; e Faculty of Science, Charles University in Prague, Viničná 7, 128 44 Prague, Czech Republic

## Abstract

Production of nitro compounds has only seldom been recorded in arthropods. The aliphatic nitroalkene (*E*)-nitropentadec-1-ene (**NPD**), identified in soldiers of the termite genus *Prorhinotermes*, was the first case documented in insects in early seventies. Yet, the biosynthetic origin of **NPD** has long remained unknown. We previously proposed that N**PD** arises through the condensation of amino acids glycine and/or l-serine with tetradecanoic acid along a biosynthetic pathway analogous to the formation of sphingolipids. Here, we provide a metabolomics and transcriptomic data of the *Prorhinotermes simplex* termite workers and soldiers. Data are related to **NPD** biosynthesis in *P. simplex* soldiers. Original metabolomics data were deposited in MetaboLights metabolomics database and are become publicly available after publishing the original article. Additionally, chemical synthesis of biosynthetic intermediates of **NPD** in nonlabeled and stable labeled forms are reported. Data extend our poor knowledge of arthropod metabolome and transcriptome and would be useful for comparative study in termites or other arthropods. The data were used for de-replication of **NPD** biosynthesis and published separately (Jirošová et al., 2017) [1].

**Specifications table**

**Table:** 

Subject area	*Chemistry, Biology*
More specific subject area	*Biosynthesis of lipids, sphingosine pathway, mass spectrometry*
Type of data	*text file, graphs, figures, links to raw data*
How data was acquired	*Mass spectrometry with C18 column separation, Q-Exactive plus (Thermo), RNAseq on an Illumina HiSeq. 2500 Genome Analyzer platform using paired-end* (2×100 bp) *reads*
Data format	*Filtered, analyzed, link to raw files*
Experimental factors	*Termite workers and soldiers, different parts of termite body, different ages of termite soldiers*
Experimental features	*RNA of soldier abdominal cavity without gut, soldier legs, soldier frontal glands, and worker abdominal cavity without gut was extracted and RNAseq on Illumina platform. Data processing and normalization was performed in a standard way.*
	*Methanol/dichlormethane extracts of workers and six developmental soldier stages (presoldier, -1 day, 0 day, 1 day, 3 days, 7 days) were separated on C18 column and eluted compounds detected in Q-Exactive tandem mass spectrometer in MS and MS/MS mode.*
Data source location	*Prague 6, Prague, Czech Republic, laboratory colony 50.1054925N, 14.3908131E*
Data accessibility	*Raw LC/MS data are deposited in Dryad repository under doi:10.5061/dryad.87s0dp4 and RNAseq data are available at European Nucleotide Archive (ENA) under study numberPRJEB18967 (https://www.ebi.ac.uk/ena/data/view/PRJEB18967)*

**Value of the data**

• LC–MS and LC–MS/MS data exemplified in Fig. 2 and raw data submitted under xxx in Dryiad will be useful for comparative study on metabolomics of other termite species as well as for other arthropods.
• Transcriptomic data exemplified in Fig. 3, Fig. 4, Fig. 5 and raw data submitted under xxx in Dryiad will be useful for comparative study on transcriptome of other termite species as well as for other arthropods and we welcome other groups using our data and building a cooperation in future.
• Chemical synthetic methods here used can be extended to other aminoalcohols, aminoketones and nitro compounds with different chains and substitution pattern.

## 1 Data

**Fig. 1:**
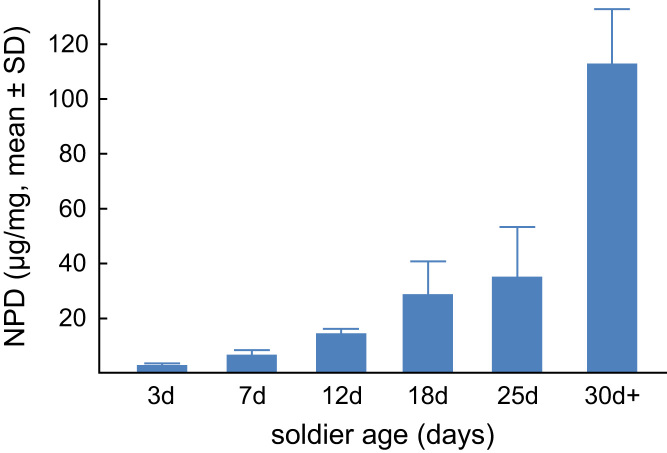
Time dynamics in NPD production in young soldiers based on GC-FID quantifications in soldier homogenates, related to the body mass of soldiers. Seven replicates (individual soldiers) were analyzed for each age class.

Detailed description of chemical synthesis of standards and incubation probes used as biosynthetic precursors for NPD biosynthesis pathway elucidation (Fig. 1, Fig. 6, Fig. 7).

**Fig. 2:**
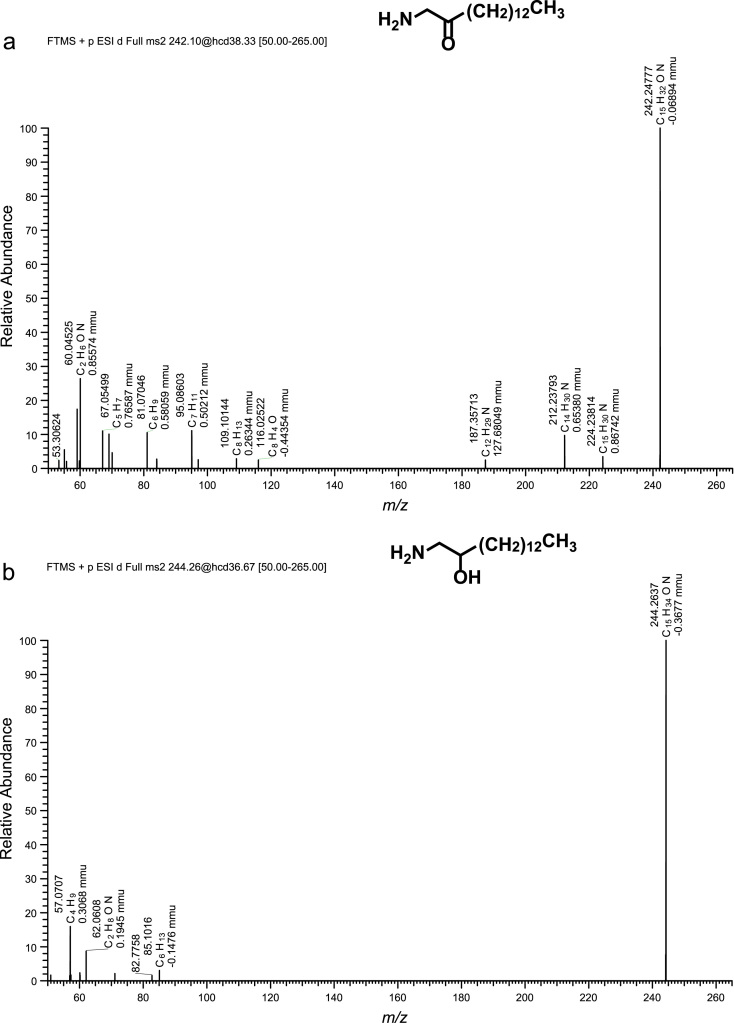

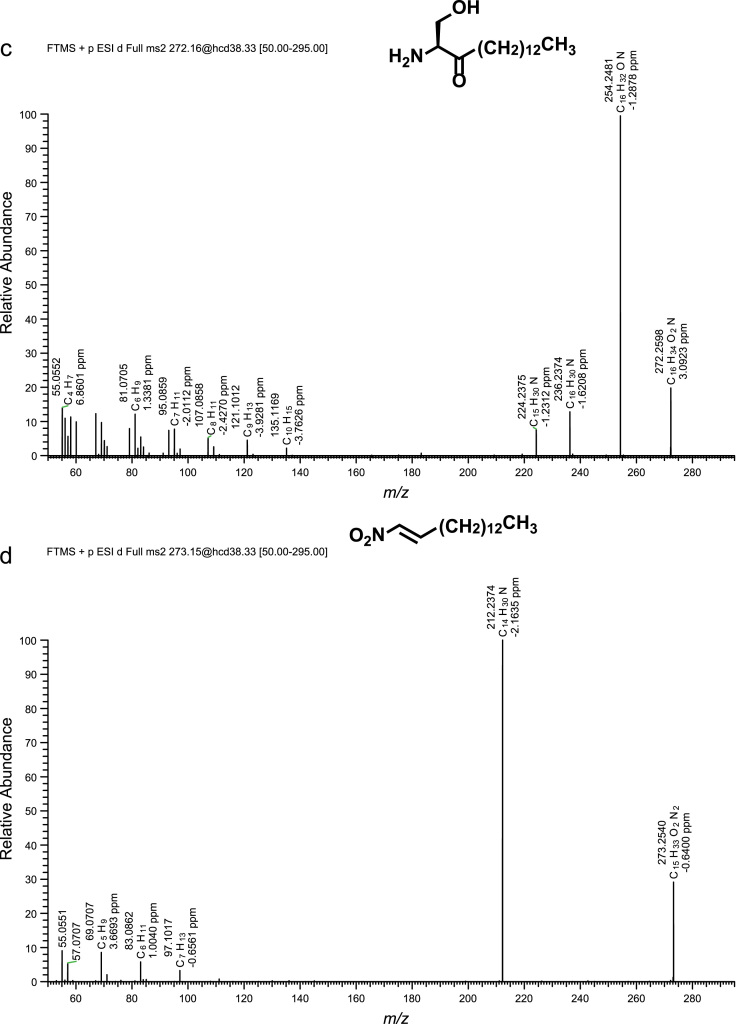

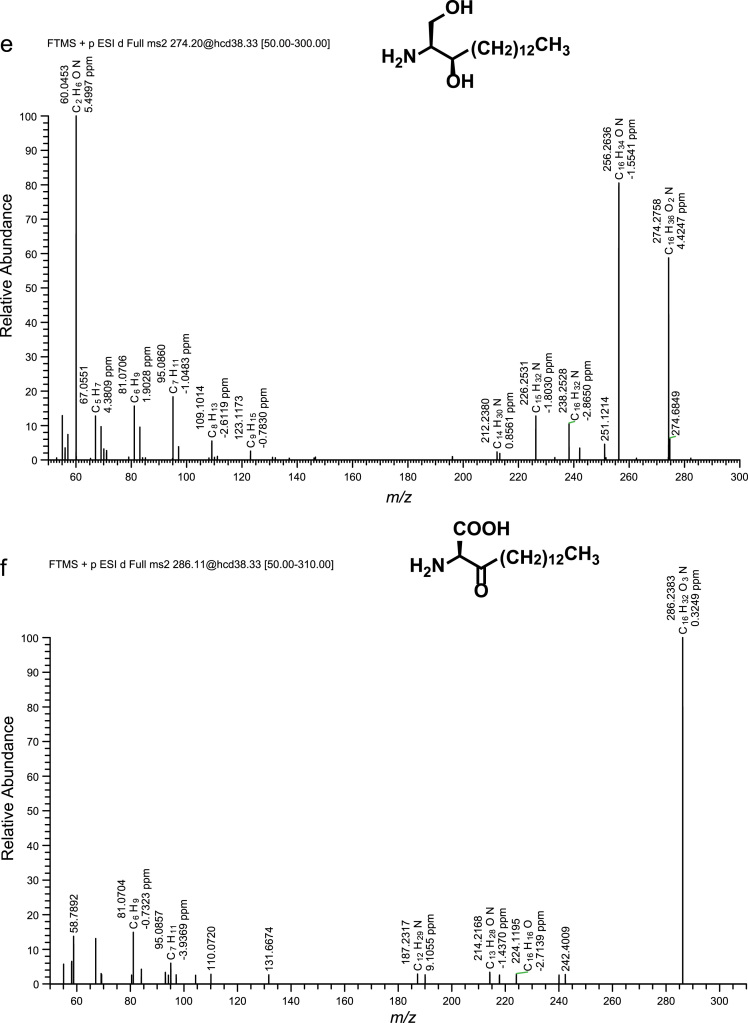

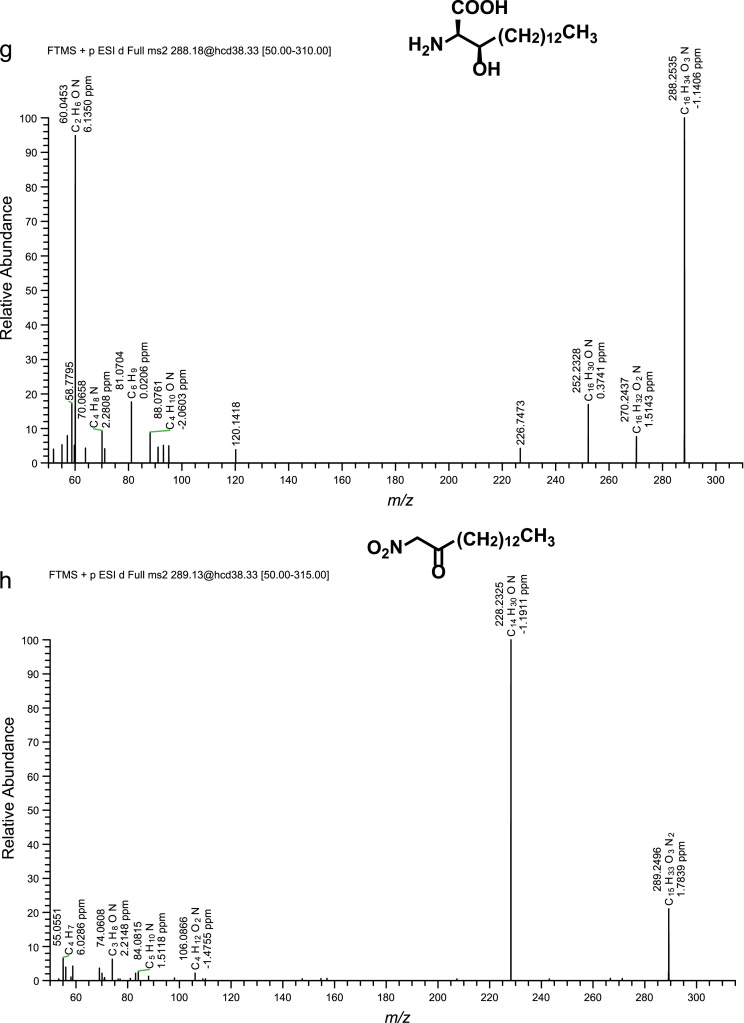

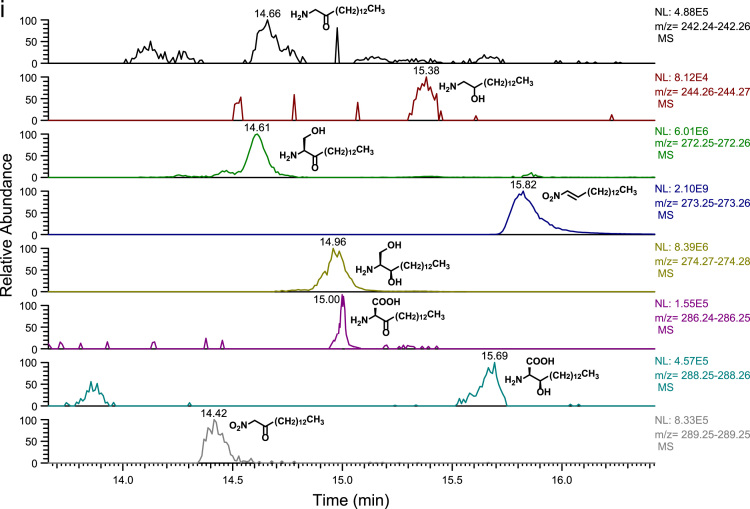
(a)–(h) CID spectra. (i) A section of SIMs traces of identified metabolites. For details see reference [1].

**Fig. 3:**
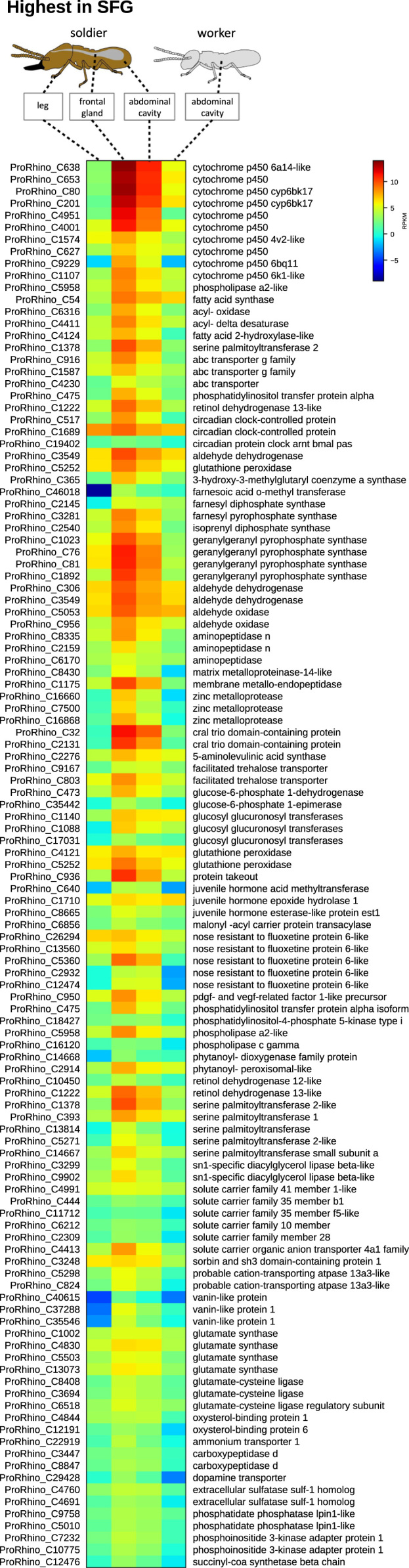
Heat map of genes which display highest expression levels in the *P. simplex* soldier frontal gland tissue. Origin of tissue material for RNAseq is depicted above the respective sample columns. The map is based on log2-transformed RPKM values (blue represents weakly-expressed genes, and red represents strongly-expressed genes).

**Fig. 4:**
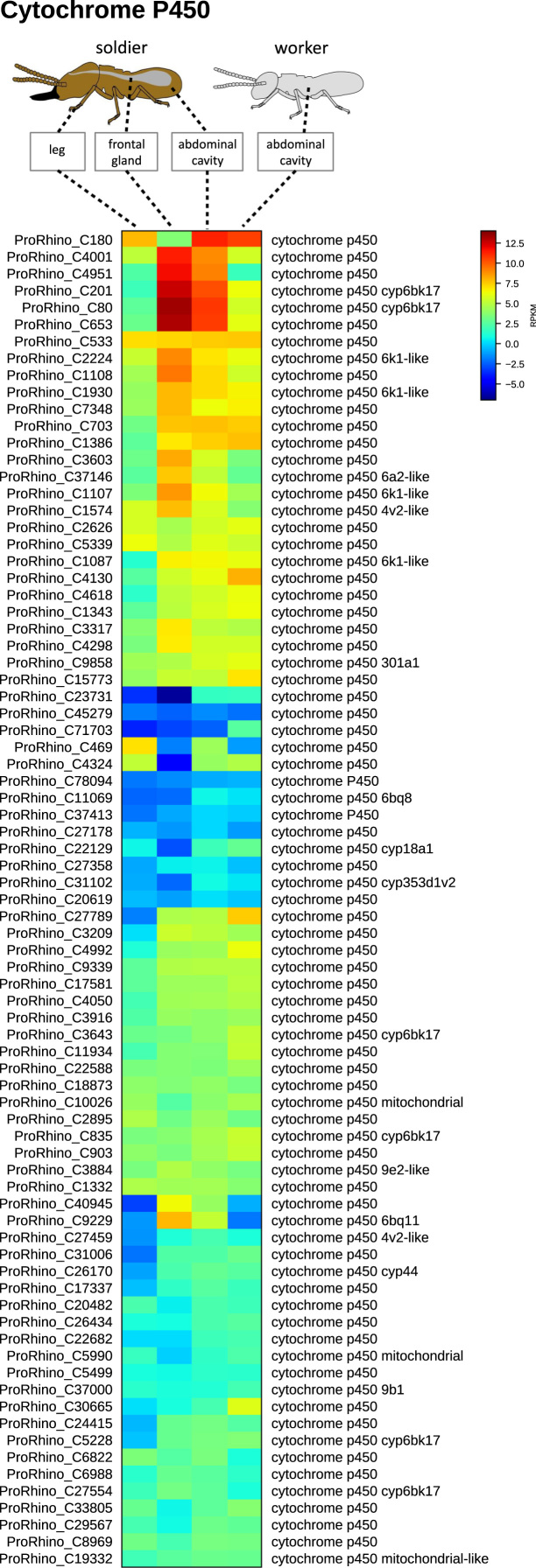
Heat map of cytochrome P450 genes identified in the *P. simplex* transcriptome. Origin of tissue material for RNAseq is depicted above the respective sample columns. The map is based on log2-transformed RPKM values (blue represents weakly-expressed genes, and red represents strongly-expressed genes).

**Fig. 5:**
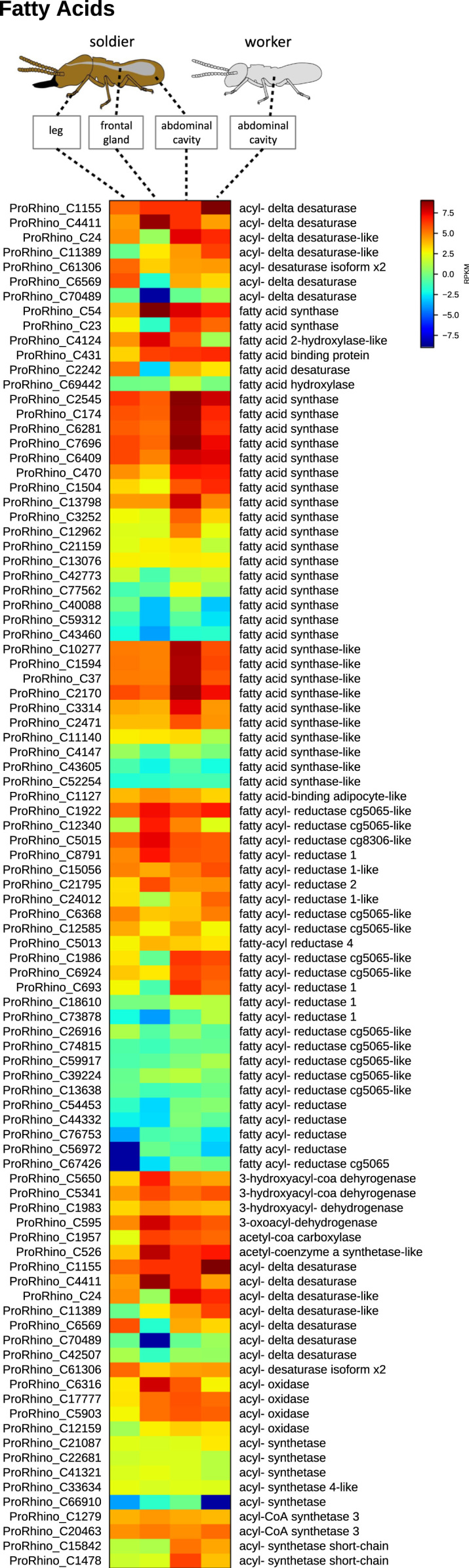
Heat map of fatty acid biosynthesis-related genes identified in the *P. simplex* transcriptome. Origin of tissue material for RNAseq is depicted above the respective sample columns. The map is based on log2-transformed RPKM values (blue represents weakly-expressed genes, and red represents strongly-expressed genes).

**Fig. 6:**
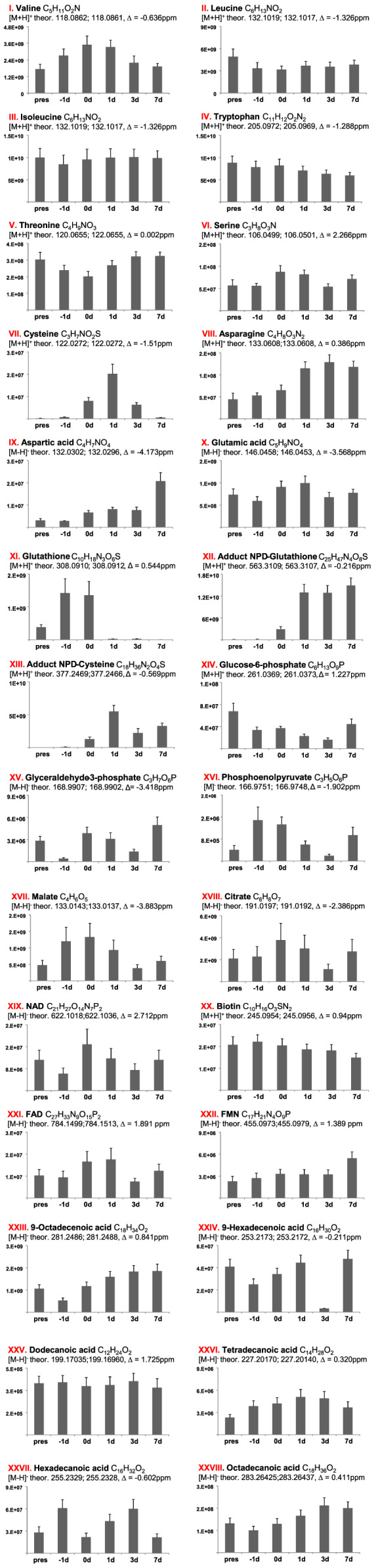
Time dynamics of identified metabolites in six developmental stages of *P. simplex* soldiers based on UPLC–MS quantifications in soldier extracts. Three replicates (pooled five soldiers) were analyzed for each age class.

**Fig. 7:**
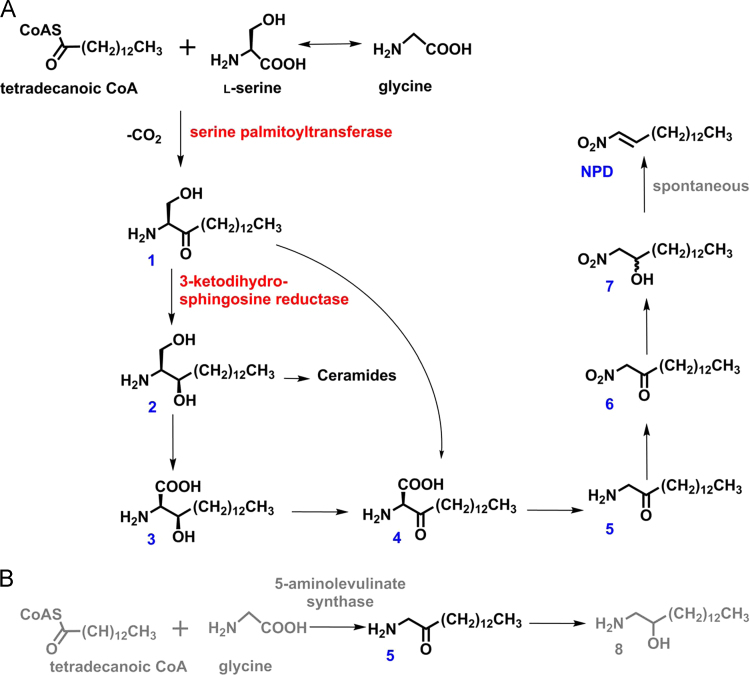
NPD putative biosynthetic pathway. (A) The main route, (B) Alternative way for aminoketone (**5**) biosynthesis.

## 2 Experimental design, materials and methods

### 2.1 Synthesis of intermediates and metabolites

The following standards of precursors and putative intermediates were synthesized: 1-nitropentadecan-2-one, 1-nitropentadecan-2-ol 1-aminopentadecan-2-ol and **NPD**. The following analogs labeled with deuterium at 13 carbon atoms of the aliphatic chain were synthesized: D_27_-(2*S*,3*R*)-2-aminohexadecane-1,3-diol (D_27_-**2**), D_27_-(2*R*,3*R*)-2-amino-3-hydroxyhexadecanoic acid (D_27_-**3**), D_27_-1-nitropentadecan-2-ol (D_27_-**7**), D_27_-1-aminopentadecan-2-ol (D_27_-**8**) and D_27_-(*E*)-1-nitropentadec-1-ene (D_27_-**NPD**) using published procedure [2], [3], [4].

### 2.2 Sample preparation

For UHPLC–ESI–MS/MS system (Q-Exactive, Thermo) analyses, soldiers and control workers were homogenized in a glass teflon homogenizer and extracted in dichloromethane:methanol (2:1, v/v) (5 individuals per sample in 250 µl) for 40 min at room temperature. After sonication, the samples were filtered through extracted cotton wool in glass Pasteur pipettes and 10 μL was injected into UPLC. For GC-FID analyses, individual soldiers were put into glass vials containing 50 μL of dichloromethane, homogenized and sonicated for 5 min. The liquid fraction was injected into a GC.

### 2.3 UHPLC–ESI–MS

Samples were analyzed on Ultimate 3000 series RSLC (Dionex, Sunnyvale, CA, USA) system coupled to a Q-Exactive Plus Hybrid Quadrupole-Orbitrap Mass Spectrometer (Thermo Fisher Scientific, Bremen, Germany) equipped with an ESI source. The separation was performed on Acclaim RSLC column (2.1 mm×150 mm, C18, 2.2 µm particles with 120 Å pore diameter, Thermo Scientific) using water with 0.1% formic acid as solvent A and acetonitrile with 0.1% formic acid as solvent B at 300 µl flow rate. The gradient was as follows: 0–3 min, 0% B; 3–15 min, 60% B; 15–30 min, 100% B; 30–38 min, 100% B; 38–38.1 min, 0% B; 38.1–45 min, 0% B. Each sample run was separated with a short gradient wash run: 0–3 min, 0% B; 3–8 min, 60% B; 8–14 min, 100% B; 14–20 min, 100% B; 20–20.5 min, 0% B; 20.5–25 min, 0% B. The orbitrap mass analyzer was set to 140,000 mass resolutions at *m/z* 200 and operated in positive or negative ion mode. For tandem MS/MS, masses of interest were selected in a quadrupole using 1 Da selection window, and normalized fragmentation energy was set from 10 to 35 V. The instrument was calibrated using commercial CallMix (Thermo) prior to each sequence. Continuum spectral data of *m/z* 100–1500 mass span were collected and analyzed using Xcalibur v.3.0.63 software (Thermo Fisher Scientific).

### 2.4 Data analysis

For semi-quantification based on the retention times and mass spectra of reference compounds, **NPD** and its precursors and likely intermediates were identified in termite extracts. Several primary metabolites involved in the metabolism of the Krebs cycle and fatty acids (FAs); additionally, amino acids were identified based on accurate mass data and in some cases on CID spectra. The peaks of these compounds were integrated in Xcalibur and the area under the curve was calculated.

### 2.5 RNA-Seq and differential gene expression analysis

RNA was extracted from the following dissected tissues and body parts of *P. simplex* soldier and worker caste: 1) soldier abdominal cavity without gut (pooled tissue from 10 specimens), 2) soldier legs (10 specimens), soldier frontal glands (50 specimens), and worker abdominal cavity without gut (10 specimens) stored in TRIzol (Invitrogen) at −80 °C prior to RNA extraction. Total RNA was extracted using standard phenol–chloroform procedure with TRIzol according to the manufacturer's protocol (Life Technologies), followed by digestion of DNA contaminants with TURBO DNase (Ambion) at 37 °C for 1 h and subsequent RNA purification using the RNeasy Mini Kit (Qiagen) according to the manufacturer's protocol for RNA cleanup. The quantity of RNA was determined using a Nanodrop ND-1000 UV/Vis spectrophotometer (Thermo Fisher Scientific). The integrity of the RNA was verified using an Agilent 2100 Bioanalyzer and a RNA 6000 Nano Kit (Agilent Technologies, Palo Alto, CA).

Tissue-specific transcriptome sequencing of the four different RNA samples was performed with poly(A)+ enriched mRNA fragmented to an average of 150 nucleotides. Sequencing was carried out by the Max Planck Genome Center Cologne (MPGCC) on an Illumina HiSeq. 2500 Genome Analyzer platform using paired-end (2×100 bp) reads. This yielded approximately 25 million paired-end reads for each of the four samples. Quality control measures, including the filtering of high-quality reads based on the score given in fastq files, removal of reads containing primer/adaptor sequences and trimming of read length, were carried out using CLC Genomics Workbench v8.1 (http://www.clcbio.com). The *de novo* transcriptome assembly was carried out with the same software, combining all of the four RNA-Seq samples, and selecting the presumed optimal consensus transcriptome as described in Vogel et al. [5]. The resulting final *de novo* reference transcriptome assembly (backbone) of *P. simplex* contained 79,916 contigs (minimum contig size=300 bp) with a N50 contig size of 1486 bp and a maximum contig length of 27,056 bp. The transcriptome was annotated using BLAST, Gene Ontology and InterProScan searches using BLAST2GO PRO v3.1 (www.blast2go.de).

Digital gene expression analysis was carried out using CLC Genomics Workbench v8.1 to generate BAM (mapping) files, and QSeq Software (DNAStar Inc., Madison, WI, USA) was then used to remap the Illumina reads from all four samples onto the reference transcriptome followed by counting the sequences to estimate expression levels, using previously described parameters for read mapping and normalization [5].
